# Deep Functional and Molecular Characterization of a High-Risk Undifferentiated Pleomorphic Sarcoma

**DOI:** 10.1155/2020/6312480

**Published:** 2020-05-04

**Authors:** Noah E. Berlow, Catherine S. Grasso, Michael J. Quist, Mingshan Cheng, Regina Gandour-Edwards, Brian S. Hernandez, Joel E. Michalek, Christopher Ryan, Paul Spellman, Ranadip Pal, Lynn S. Million, Mark Renneker, Charles Keller

**Affiliations:** ^1^Children's Cancer Therapy Development Institute, Beaverton, OR 97005, USA; ^2^Electrical and Computer Engineering, Texas Tech University, Lubbock, TX 79409, USA; ^3^Division of Hematology-Oncology, University of California, Los Angeles, CA 90095, USA; ^4^The Jackson Laboratory, Bar Harbor, ME 04609, USA; ^5^Department of Pathology & Laboratory Medicine, UC Davis Health System, Sacramento, CA 95817, USA; ^6^Department of Epidemiology and Biostatistics, University of Texas Health Science Center San Antonio, San Antonio, TX 78229, USA; ^7^School of Medicine, Oregon Health and Science University, Portland, OR 97239, USA; ^8^Department of Radiation Oncology, Stanford University, Stanford, CA 94305, USA; ^9^Patient-Directed Consultations, San Francisco, CA 94116, USA; ^10^Department of Family Medicine, University of California San Francisco, San Francisco, CA 94143, USA

## Abstract

Nonrhabdomyosarcoma soft-tissue sarcomas (STSs) are a class of 50+ cancers arising in muscle and soft tissues of children, adolescents, and adults. Rarity of each subtype often precludes subtype-specific preclinical research, leaving many STS patients with limited treatment options should frontline therapy be insufficient. When clinical options are exhausted, personalized therapy assignment approaches may help direct patient care. Here, we report the results of an adult female STS patient with relapsed undifferentiated pleomorphic sarcoma (UPS) who self-drove exploration of a wide array of personalized Clinical Laboratory Improvement Amendments (CLIAs) level and research-level diagnostics, including state of the art genomic, proteomic, *ex vivo* live cell chemosensitivity testing, a patient-derived xenograft model, and immunoscoring. Her therapeutic choices were also diverse, including neoadjuvant chemotherapy, radiation therapy, and surgeries. Adjuvant and recurrence strategies included off-label and natural medicines, several immunotherapies, and N-of-1 approaches. Identified treatment options, especially those validated during the *in vivo* study, were not introduced into the course of clinical treatment but did provide plausible treatment regimens based on FDA-approved clinical agents.

## 1. Introduction

Nonrhabdomyosarcoma soft-tissue sarcomas (NRSTSs) are a collection of 50+ soft-tissue tumors occurring from infancy to geriatric ages. Due to rarity of each subtype and limited preclinical research models, individual NRSTS subtypes remain underserved from the basic science and preclinical investigation perspective. The few established clinical trials often treat NRSTS as a group rather than a spectrum of individual diseases (e.g., NCT02180867 and NCT02267083). Distant metastasis is the major cause of death in NRSTS [1]. Inconsistent response to chemotherapy makes complete surgical resection an essential aspect of NRSTS therapy.

A retrospective study of adult soft-tissue sarcoma patients analyzed survival following surgical resection based on a modified Union for International Cancer Control (UICC) tumor-node-metastasis (TNM) criterion. The two criterion are R0M (resection with clear margins including satellite nodules and proliferation contours) and R1M (resection with infiltrated margins including satellite nodules and proliferation contours). Thirty-eight percent of NRSTS patient surgeries were classified as R1M (incomplete resection). When segmented by R0M/R1M status, the 5-year local recurrence-free survival (LRFS) following R0M/R1M surgery was 92%/63% (*p*=0.001) [1], the 5-year disease-free survival (DFS) was 69%/32% (*p* < 0.001) [1], and the 5-year metastasis-free survival (MFS) was 75%/43% (*p*=0.007) [1]. Among patients with initially unresectable tumors who received chemotherapy or local radiotherapy, the 5-year survival rate following local recurrence/progression is 9% [2, 3]. Statistics for UPS show the limitations of current clinical standard of care, where 38% of NRSTS patients expect ∼30% difference in 5-year survival based on surgical outcomes alone. New therapies are critically needed to address unresectable tumors, residual local disease, and distant metastasis of tumors to improve outcomes for NRSTS patients.

The NRSTS *undifferentiated pleomorphic sarcoma* (UPS), formerly known as malignant fibrous histiocytoma (MFH), is an aggressive malignant soft-tissue or bone sarcoma arising both distally and proximally [4, 5]. UPS is the 4^th^ most common soft-tissue sarcoma with approximately 3 cases per 100,000 persons/year [6] and occurs across the age spectrum, afflicting pediatric, young adult, and adult patients, although UPS occurs most commonly in 60–80-year-old patients. UPS is often characterized by presence of a tumor mass resulting in swelling, pain, cancer-induced pathological bone fracture, and additional systemic features [4, 5]. UPS has both a high rate of recurrence and significant metastatic burden (distant metastases more likely than regional, with lungs as the most frequent metastatic site) [4, 5]. Overall 5-year survival for head and neck UPS tumors is 48% versus 77% for trunk and extremity UPS cases [4, 5]. Here, we present the case of an adult female patient with UPS, with a focus on multiple approaches explored for personalization of therapy. A timeline of treatments and events is provided in Supplemental Figure 1.

## 2. Results

### 2.1. Clinical Presentation

Our index case is a 61-year-old female with a history of bilateral breast cancers, basal cell carcinoma, and multiple lipomas. She presented with a swollen, painful right posterior thigh found on imaging to contain a 14 cm mass. Tru-cut® biopsy revealed a high-grade (FNLCC grade 3) undifferentiated sarcoma. No evidence of metastases was found on additional imaging, and she was assessed as stage III, T2bN0M0.

### 2.2. Initial Treatment

The patient began with neoadjuvant therapy at her local cancer center, consisting of one cycle of doxorubicin and ifosfamide, followed by 2 cycles of ifosfamide with concurrent radiation therapy which elicited approximately 30% tumor reduction. A wide excision surgical plan that would include part of her femur was proposed. To avoid sacrifice of bone and the sciatic nerve, she opted for six additional rounds of higher dose chemotherapy consisting of 4 cycles of doxorubicin and ifosfamide followed by two cycles of gemcitabine and docetaxel which resulted in approximately 67% tumor size reduction but with increasing toxicities. After 9 months of neoadjuvant chemotherapy, the patient underwent bone and sciatic nerve-sparing surgery at a specialized cancer center. Clear margins were achieved. Pathology revealed a 5 cm pleomorphic spindle cell sarcoma, the majority of which had a low mitotic rate, except for an embedded 2 cm area of higher grade disease which showed up to 58 mitoses/hpf. Treatment effect was evident from histopathology. Three additional cycles of adjuvant chemotherapy with doxorubicin and ifosfamide were recommended, but the patient only tolerated one cycle.

### 2.3. Exploration of Therapeutic Options

During the second round of neoadjuvant therapy, the patient came to realize her risk of recurrence was considerable and decided to expand her knowledge of soft-tissue sarcomas and explore the state of sarcoma research, trials, and expertise at institutions outside her treating institution. Adjuvant therapy clinical trials that might apply to her case were sought, but none were available. As such, she decided to use her resources and professional management experience to investigate her own therapeutic regimens and research resources.

The patient enlisted the ongoing help of an experienced medical advocate physician (coauthor M. R.) to provide her with information relevant to her disease type and help her explore potential strategies to overcome limited information and research available for her comparatively rare cancer. The patient subsequently pursued numerous nonstandard therapeutic options in the pursuit of effective and durable treatment response. To explore nonstandard therapy options, tumor tissue from surgical resection was sent to multiple laboratories for analysis, hoping that more precise and less toxic adjuvant therapy recommendations would emerge.

### 2.4. Commercial *Ex Vivo* Chemosensitivity Assays

Live surgically excised tumor tissue was processed to create a primary cell culture which was then exposed to various single agent and combination chemotherapy and/or targeted therapy drugs associated with sarcoma. Two independent commercial laboratories (Rational Therapeutics and Weisenthal Lab) tested drug efficacy on the basis of programmed cell death chemosensitivity using similar laboratory methods. Commercial chemosensitivity testing identified, with interlaboratory concordance (4 of 8 tested agents with identical interpretation, Supplemental Table 1), several drugs to which the tumor might be sensitive, including vorinostat, interferon-alfa, dacarbazine, oxaliplatin, *Serratia marcescens* (Coley's toxin), artemisinin, phenylbutyrate, the combination of cisplatin and gemcitabine, and the combination of vinorelbine and lapatinib (Supplemental Table 1). Resistance or low sensitivity was predicted for drugs the patient had previously been treated with including doxorubicin, ifosfamide, gemcitabine, and taxotere (Supplemental Table 1). Based on the ex vivo chemosensitivity results and quality-of-life challenges from previous rounds of high-dose chemotherapy, the patient elected to not pursue further use of the chemotherapy agents predicted to be resistant by the chemosensitivity assays. A separate research-level sensitivity assay performed at Oregon Health and Science University (OHSU) is presented in a later section. Concordance between the commercial *ex vivo* assays and the research-level sensitivity assay is provided in Supplemental Table 2.

### 2.5. Immunotherapy

The patient also pursued cancer immunotherapy, focusing on maintenance immunotherapy protocols presented at the Annual Meetings of the American Association of Cancer Research [7]. Subsequently, a local integrative physician prescribed the patient a modified Recchia protocol using low-dose subcutaneous interleukin 2 (1–1.2 million units) and oral cis-retinoic acid (0.5 mg/kg) three times weekly, every other week. The patient was able to obtain and use maintenance immunotherapy based on a subcutaneous thymic stimulator called thymosin-alpha (1.6 mg twice weekly) [8] which has been approved outside the US. She also arranged to have leukapheresis and to receive a personalized dendritic cell vaccine [9].

### 2.6. Additional Wellness Approaches

Terrain testing was performed to identify potentially actionable blood levels of various vitamins, minerals, inflammatory markers, coagulation markers, and immune markers, which lead to supplementation with specific vitamins (particularly vitamin D), curcumin, green tea extract, omega-3 fatty acids, and mushroom extracts. The patient also optimized her sleep schedule and engaged in various Mind-Body approaches, including an aggressive physical therapy and training program to recover from the surgery and to improve her physical health.

### 2.7. Xenograft Model Development

**Figure 1:**
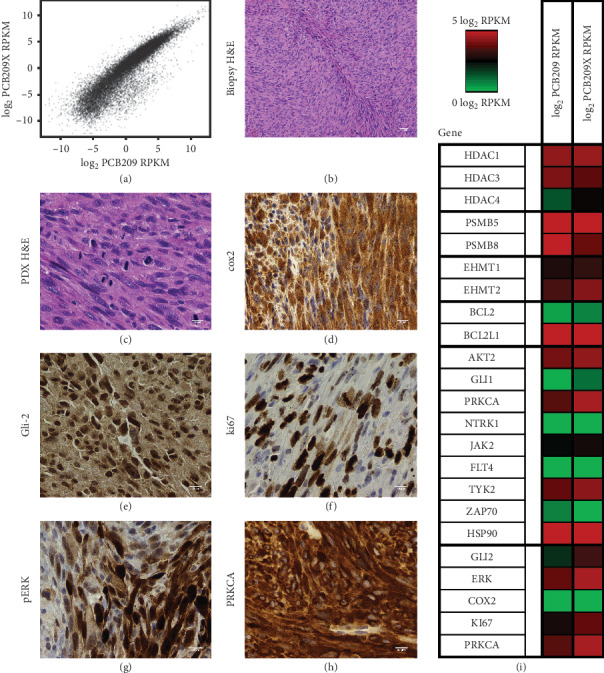
RNA-seq and histology of PCB-209. RNA-seq data for PTIM identified targets and multiple staining images of PCB-209. RNA-seq and histology data were used to partially guide cohorts of the *in vivo* PDX trial. (a) RNA-seq expression of PCB-209 vs. PCB-209 PDX. (b) H&E stain. (c) PDX H&E stain. (d) Staining for COX2. (e) Staining for GLI2. (f) Staining for ki67. (g) Staining for pERK. (h) Staining for PRKCA. (i) RNA-seq RPKM reported in log_2_ format for targets identified by the PTIM analysis informed with RNA-seq data. Red indicates high expression, and green indicates low expression. The left column is the expression for the original cell culture, and the right column is the expression for the culture created from the xenograft mice.

Tissue samples from the patient's tumor (denoted PCB-209) were sent to our laboratory following surgical resection of tumor tissue. From the live tissue sample, a primary cell culture was created, a research-level targeted agent sensitivity panel was performed, and whole genome and transcriptome sequencing was performed through sequencing partners. Tumor tissue was shared with the Jackson Laboratory (JAX), where a patient-derived xenograft (PDX) was established in a mouse (PDX model TM00381) to enable downstream assessment of potential chemotherapeutic and targeted therapy drugs. Log-scaled expression data showed high correlation (*ρ* = 0.9432) between PCB-209 primary tumor and PCB-209 PDX tumor (Figure 1(a)), and hematoxylin and eosin stain analysis of primary tumor tissue versus PDX tumor tissue (Figures 1(b) and 1(c)) showed consistent histology before and after engraftment. Representative IHC images for PCB-209 show presence of proteins of interest from morphoproteomic analysis or published UPS biology [10, 11] (Figures 1(d)–1(h)).

### 2.8. Research-Level Chemosensitivity Assay

Primary PCB-209 tissue was cultured and subsequently screened with the PPTI Version 2.1 single agent chemical screen. Due to slow primary culture cell growth, PCB-209 primary culture was screened on only 19 of 60 agents (Supplemental Table 3). Tumor tissue explanted from the established PCB-209 PDX mouse model was cultured and also screened with the PPTI Version 2.1 single agent chemical screen (Supplemental Table 3). Comparison of primary culture vs. PDX response data showed sensitivity to CDK9 inhibition (alvocidib) in the primary culture; sensitivity to Mao/autophagy pathway inhibition (quinacrine) and GLI1/2 inhibition (GANT 61) in the PDX culture; and concordance in sensitivity to proteasome inhibition (carfilzomib) and ALK/MET inhibition (crizotinib). Moderate crizotinib sensitivity was consistent across the research-level screen and the commercial screen. Chemical screening identified a total of 16 compounds with activity for the PCB-209 PDX cell culture.

### 2.9. Genomic and Transcriptomic Profiling

DNA and RNA isolated from PCB-209 primary tumor tissue were sent for whole genome and whole transcriptome sequencing. Genomic profiling was largely by Next Generation Sequencing (NGS), performed by Foundation Medicine, Caris, and the Genomic Profiling Shared Resource at OHSU.

Ten actionable alterations were noted, including several *FGF* genes, *CCND1* and *EMSY* amplified genes, *ALK* gain, *TP53* and *CDKN2A/B* losses, and a mutation of *ATRX* (F529fs). No FDA-approved drugs or sarcoma-specific clinical trials matched the identified gene abnormalities. NGS also identified 12 variants of unknown significance. At the time, the genomic profiling was performed, and status of microsatellite genes was not reported. Nonetheless, all three tested microsatellite-related genes (*MSH2*, *MSH6*, and *PMS2*) were intact.

Because the genomic profiling results were not clinically actionable, the patient commissioned a literature review to examine published research on the identified gene abnormalities and abnormalities in the variant genes in general (*i.e.*, not for the patient's specific mutation) for all 22 variant genes to identify possible off-label medications or natural products with published activity. The majority of variant genes had associated citations from cell line-based experiments, which were used to further personalize her adjuvant supplement plan.

**Figure 2:**
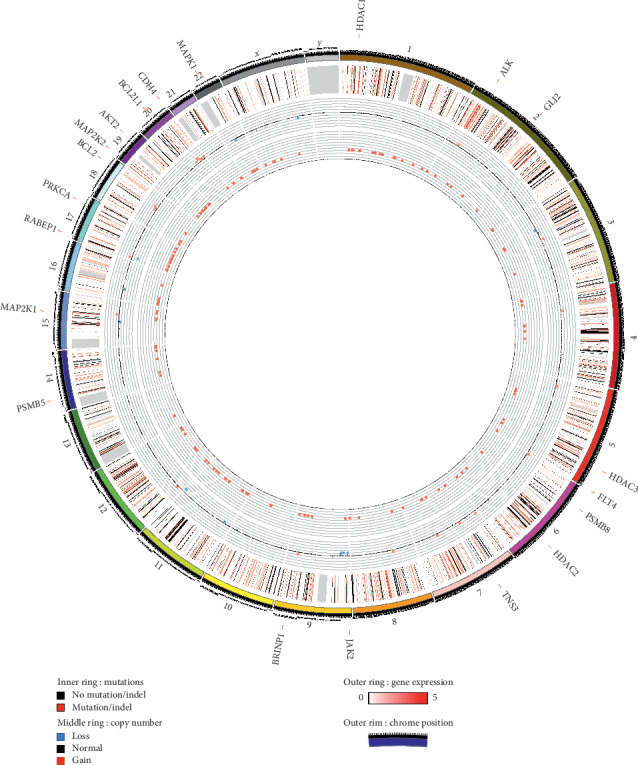
Circos plot of PCB-209 RNA sequencing and whole genome sequencing data. The outermost data circle represents log_2_-scaled gene expression (green represents negative, red represents positive) for genes with identified mutations or copy number variations. The middle circle represents genes with identified mutations or indels (black) or lack thereof (white). The innermost circle represents copy number variations (pink is amplification, light blue is deletion, and white is no variation). Genes which carry both mutations and amplifications (*TNS3*, *BRINP1*, *RABEP1*, and *CDH4*) are written in blue around the circle, as are genes relevant to *in vivo* studies (*HDAC1*, *ALK*, *GL1*, *HDAC3*, *FLT4*, *PSMB8*, *HDAC2*, *JAK2*, *PSMB5*, *MAP2K1*, *PRKCA*, *BCL2*, *MAP2K2*, *AKT2*, *BCL2L1*, and *MAPK1*).

RNA isolated from PCB-209 primary tumor tissue and PCB-209 PDX explant tissue was also sent for transcriptome sequencing to determine overall gene expression and identify systemic changes in gene expression following PDX establishment. Genomic and transcriptomic data are summarized in Circos plot format (Figure 2) and in tabular format (Supplemental Table 4).

### 2.10. Morphoproteomic Immunohistochemistry Analysis

Morphoproteomic analysis (immunohistochemistry panel) qualifies and quantifies protein expression from formalin fixed, paraffin embedded (FFPE) tissue slides to identify patient-specific treatment options. Morphoproteomic analysis by immunohistochemistry was performed by the Brown Laboratory at the University of Texas Health Science Center Houston [12] and focused on the higher-grade portion of the tumor which showed IGF1R and PRKCA as the principal upstream signal transducer drivers and mTOR as a downstream effector. Areas of high protein expression of COX2, SIRT1, STAT3, HIF1A, PPARG, NES, CD133, GLI2, and SPARC were also identified. Recommendations included albumin-bound paclitaxel and off-label agents or natural medicines including metformin, COX2 inhibitors, vorinostat, and melatonin. Morphoproteomic analysis recommendations referenced cell-line and animal studies. The patient chose to forego the chemotherapies and HDAC inhibitor but did begin on metformin (850 mg/day), a COX2 inhibitor, and melatonin (20 mg/night). Morphoproteomic analysis results are presented in Supplemental Table 5.

### 2.11. Probabilistic Target Inhibition Map Modeling

**Figure 3:**
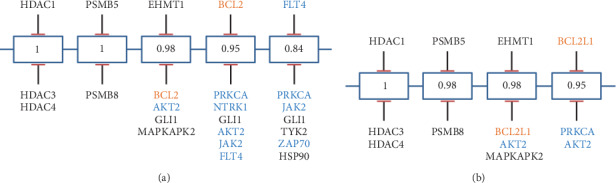
Probabilistic Target Inhibitor Map (PTIM) model of the PCB209 xenograft-derived chemical screen. Orange text indicate targets inhibited by ABY-737; blue text indicates targets inhibited by midostaurin. (a) Pediatric preclinical testing initiative (PPTI) 60 chemical screen informed PTIM. (b) RNA-seq + PPTI chemical screen informed PTIM. PTIM models were used to guide a portion of the larger PDX *in vivo* trial shown in Figure 2. Numbers listed in the individual boxes represent scaled sensitivity values, 1 being a highly sensitive target combination and 0 being an ineffective/resistant target combination.

Probabilistic Target Inhibition Map (PTIM) modeling [13–18] integrates patient-specific chemical screening data with matched genomic and transcriptomic sequencing data to design personalized drug combinations. PTIM modeling identifies drug combinations where the individual agents may not slow or stop tumor growth but in combination will be synergistic and slow tumor growth. PTIM modeling analysis of PCB-209 PDX chemical screening data (Figure 3(a)) with integrated PCB-209 genomic and transcriptomic sequencing data (Figure 3(b)) identified multitarget explanations for *in vitro* chemical screen sensitivities and was used to predict an efficacious drug combination, ABT-737 (BCL2 inhibitor) with midostaurin (multikinase inhibitor). PTIM modeling is independent of morphoproteomic analysis and integrated only the PCB-209 PDX chemosensitivity assay data and genomic and transcriptomic profiling data (Supplemental Table 6).

### 2.12. Patient-Derived Xenograft Therapy Selection and Validation

**Figure 4:**
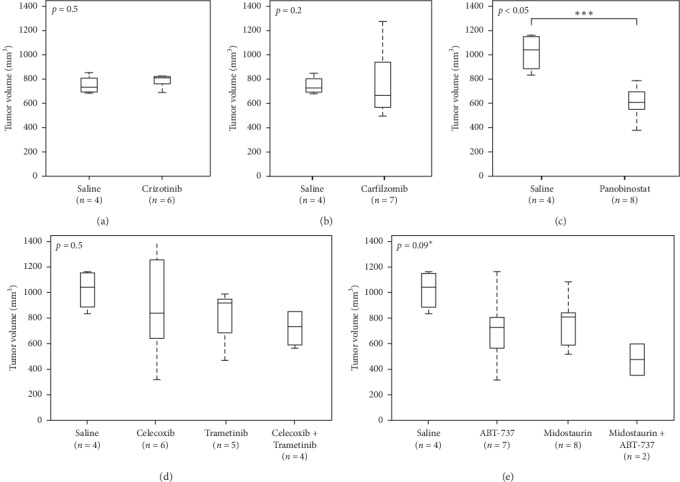
PCB-209 xenograft-derived *in vivo* trial results. Mean tumor volume of all treatment cohorts of the Jackson Lab-based PCB-209 patient-derived xenograft (PDX) model *in vivo* preclinical studies. (a) Tumor growth of crizotinib-treated PDX mice (*n* = 6) versus control (*n* = 4). (b) Tumor growth of carfilzomib-treated PDX mice (*n* = 7) versus control (*n* = 4). (c) Tumor growth of panobinostat-treated PDX mice (*n* = 8) versus control (*n* = 4). (d) Tumor growth of celecoxib- (*n* = 5), trametinib- (*n* = 5), and celecoxib + trametinib-treated (*n* = 4) PDX mice versus control (*n* = 4). (e) Tumor growth of ABT-737 (*n* = 7), midostaurin (*n* = 8), and ABT-737 + midostaurin-treated PDX mice (*n* = 2) versus control (*n* = 4). The *p* value was calculated without the midostaurin + ABT-737 due to low sample population, noted by the asterisk. The low number of models for the combination experiment is due to microbiological considerations at the Jackson Laboratory. In (a–e), the endpoint was treatment days 30–32.

The patient-derived xenograft model established from the patient's tumor was used to perform *in vivo* testing of multiple potential treatment options. Results from *in vivo* testing are presented as tumor volumes 21 days following initiation of treatment, selected as the experimental endpoint (Figure 4).

#### 2.12.1. Whole Genome Sequencing

Whole genome sequencing identified several nonactionable variants, including amplification in the *ALK* gene. While not specifically linked to *ALK* amplification, PCB-209 showed moderate sensitivity to crizotinib *in vitro* (Supplemental Table 3); thus, the ALK-inhibitor crizotinib was selected for *in vivo* PDX validation, which showed no statistically significant slowing of tumor growth versus control (*p*=0.5, Figure 4(a)).

#### 2.12.2. Chemical Screening

Two clinically available compounds with the lowest absolute IC_50_ values (panobinostat, pan-HDAC inhibitor, and carfilzomib, proteasome inhibitor) were selected from the research-level chemosensitivity assay for *in vivo* PDX validation. Panobinostat also showed efficacy on the PCB-209 primary culture (Supplemental Table 3). Both panobinostat and carfilzomib inhibit targets were found to be expressed in both primary and PDX tumor tissue (Figure 1(i)). Carfilzomib showed no statistically significant slowing of tumor growth (*p*=0.2, Figure 4(b)), while panobinostat showed statistically significant slowing of tumor growth (*p* < 0.05, Figure 4(c)).

#### 2.12.3. Immunohistochemistry Analysis

 Immunohistochemistry-based analysis results and evidence of existing drug synergy [19] motivated the selection of celecoxib (COX2 inhibitor) and trametinib (a MEK inhibitor not identified by morphoproteomic immunohistochemistry (IHC) profiling) for *in vivo* validation. The celecoxib and trametinib combination showed a possible *in vivo* effect but was not statistically significant (*p*=0.5, Figure 4(d)).

#### 2.12.4. Probabilistic Target Inhibitor Map Modeling

PTIM modeling of PCB-209 data guided selection of the combination of ABT-737 with midostaurin for *in vivo* validation. Individually, both ABT-737 and midostaurin were predicted to not show efficacy *in vivo* but in combination would show synergy and efficacy.

As predicted, PTIM-guided single agents did not show statistically significant slowing of tumor growth (*p*=0.148, Figure 4(e)). Due to low replicates at conclusion of the *in vivo* experiment, the PTIM-guided combination could not be analyzed for statistical significance. However, the PTIM-guided combination was tracked at the lowest tumor volume *in vivo* (*µ* = 450 mm^3^, Figure 4(e)).

### 2.13. Recurrence and Outcome

Fifteen months after her initial surgery and extending over the next approximately 18 months, imaging by PET/CT (less so physical examination) showed soft-tissue changes in her thigh strongly suggestive of local recurrence, leading to several additional surgical procedures. Three times, only benign tissue changes were found, but twice malignant cells were discovered. Notably, histochemical assessment in both the benign sites and recurrence sites showed considerable immune activation (in the malignant tissue, there were up to 49 CD8+ tumor infiltrating lymphocytes per high powered field). Finally, approximately 3.5 years after her initial diagnosis, the disease became broadly invasive into her upper leg, requiring right leg amputation. Within weeks, rapidly growing disease was then found in her pelvis and lower abdomen. A brief course of off-label ipilimumab and pembrolizumab was attempted, but the patient soon died.

The Probabilistic Target Inhibitor Map (PTIM) model presented in Figure 3 captures 3 cohorts of the trial: the first PTIM block identifies panobinostat (panel C), a highly selective HDAC inhibitor, as a viable treatment option. Block 2 identifies carfilzomib, a proteasome inhibitor, as a viable option. Block 3 in the RNA-seq-informed model (Block 4 in the naive model) identifies a combination of ABT-737 (a BCL2 and BCL2L1 inhibitor) and midostaurin (an AKT2 inhibitor) as a viable treatment option. Crizotinib was identified due to PCB-209 showing *in vitro* response to crizotinib and the presence of an ALK amplification. The morphoproteomic approach identified metformin, vorinostat, melatonin, and celecoxib in conjunction with Abraxane as viable treatments, which inspired the combination of celecoxib and trametinib (a MEK inhibitor not identified by IHC profiling) for validation due to existing evidence of drug synergy [19]. Of note, vorinostat is a pan-HDAC inhibitor similar to panobinostat, which showed *in vivo* efficacy, but had IC_50_ above clinically achievable concentrations. The IHC-motivated regimen was developed independently using morphoproteomic data provided by Dr. Robert Brown of the University of Texas Medical School in Houston. Dr. Brown's recommendations were not precisely followed; thus, we term the combination of celecoxib and trametinib “IHC-motivated”. The IHC-motivated combination did not involve the PTIM modeling approach in any way.

Due to small cohort size resulting from technical considerations at the Jackson Laboratory, the ABT-737 + midostaurin cohort cannot have a complete statistical analysis generated. However, the individual drugs identified by the chemical screen and PTIM model showed *in vivo* activity, and ABT-737 + midostaurin trends to a synergistic effect. Overall, two of three treatment options were potentially relevant, with ABT-737 plus midostaurin and panobinostat able to slow *in vivo* tumor growth. Note that, for the ABT-737 plus midostaurin arm in panel (E), both drugs independently have mild capacity to slow tumor growth but in combination appear to provide greater reduction of tumor growth.

## 3. Discussion

While precision/personalized therapy selection approaches did not result in the addition of targeted therapeutic agents to the patient's clinical course, the development of patient-specific preclinical models and datasets has the potential to enable transformative personalized cancer care. Additionally, the patient elected to alter her supplemental health choices and clinical decisions based on the data provided to her through multiple assays. Median length of survival after diagnosis for high-grade UPS is 9.6 months (8.2 months to 11.4 months, 95% CI) [20]. Despite a prior history of multiple cancers, the patient survived 3.5 years following her UPS diagnosis and 16 months following relapse.

As tools for therapy selection, each model development and analysis approach carries inherent strengths and weaknesses. While a PDX model was established for PCB-209, roughly 50% of patient tumors will not engraft [21]. Additionally, the timeline for model establishment and the cost of PDX development and testing can be prohibitive to many patients, despite the high predictive accuracy (∼87%) of low-passage PDX models [21]. Molecular sequencing of patient tumors is now a fast, robust and economical option. However, approximately 60% of patients bear no actionable sequencing results [22, 23], and single drug therapy often fails to sustainably control disease progression [24]. Morphoproteomics and similar IHC-based approaches interrogate presence of proteins in tumor cells, which are often the interacting partners with targeted therapies. Unfortunately, IHC-based approaches remain lower throughput than sequencing-based approaches and thus limit the scope of analysis to a small set of genes. Functional approaches provide evidence of the intervention effect via targeted therapy agents (as in the PTIM approach) or single target knockdown of individual genes (as in high-throughput siRNA screens, not performed for the patient's tumor), which can be the critical data needed for clinical decision-making. Functional approaches rely on availability of fresh and viable tumor tissue which can result in logistical challenges or may be inaccessible if insufficient tumor tissue is available. Selection of the proper technologies and approaches for development of personalized treatment will depend on availability of time, tissue, and financial resources. Nonetheless, the need for advancing clinical use of precision and personalized medicine is overwhelming for the 600,000 patients lost to cancer every year [5].

The PCB-209 case is representative of the numerous enigmatic and high-risk UPS cases that occur every year. However, we uncovered important functional relationships and actionable targets and compounds that may be of potential value for understanding and treating UPS *en masse* for future patients. Additionally, this case report serves as a stark reminder of the lack of clinical trials or clinically validated treatment options for UPS once frontline therapy no longer controls disease. While development of additional preclinical resources and sequencing experiments will help to progress the scientific understanding of UPS, the rarity and frequency of recurrence necessitate identifying personalized treatment options for recurrent UPS whenever feasible, especially when personalization would be the only path to a viable therapy.

## 4. Methods

### 4.1. Cell Model Establishment

The human undifferentiated pleomorphic sarcoma (UPS) sample PCB209 was acquired through the Childhood Cancer Registry for Familial and Sporadic Tumors (CCuRe-FAST) tumor banking program. PCB209 tumor tissue was received 24 hours after surgical resection. Tumor tissue was minced and digested with collagenase (10 mg/ml) overnight at 4°C. Dissociated cells were cultured in RPMI-1640 media (11875085; Thermo Fisher Scientific, Waltham, MA, USA) supplemented with 10% fetal bovine serum (FBS) (26140079; Thermo Fisher Scientific) and 1% penicillin-streptomycin (15140-122; Thermo Fisher Scientific) and then incubated at 37°C/5% CO_2_. Tumor tissue was sent overnight to the Jackson Laboratory (JAX), where the PDX model of PCB209 was created. The PCB-209 PDX model was assigned to the JAX model ID TM00381. All patients enrolled in CCuRe-FAST provided informed consent, and clinical and pathologic information are maintained in a deidentified database. All aspects of the study were reviewed and approved by the Oregon Health & Science University (OHSU) Institutional Review Board (IRB).

### 4.2. PCB209 Whole Genome Sequencing Analysis

Isolated DNA was sequenced with the Illumina HiSeq 1000 in paired-end mode and quality filtered by Illumina BaseCall software. Reads were mapped to the reference human genome (NCBI build 36.1, hg18) using Bowtie [25], and probable PCR duplicates were flagged and removed. A SNV was identified as a possible variant when the variant had at least three support reads and constituted at least 10% of position coverage. Somatic variants were called if the variant had at least 8x coverage in the matched normal, and the variant occurred in less than two reads and 2% of the coverage.

Copy number variations were quantified as the segmented normalized log_2_-transformed tumor/normal exon coverage ratios. CNVer [26] was used to call genes as gained or lost, requiring an exon copy number gain or loss of 30% (ratio ≥1.3 or ≤0.7) to call the gene as gained or lost. Sequencing analysis is based on a previously published approach [27].

### 4.3. PCB209 and PCB209X RNA Deep Sequencing Analysis

The PCB209 transcriptome library was sequenced with the Illumina HiSeq 1000 in paired-end mode and filtered by Illumina BaseCall software. Reads were trimmed to 85-mers and aligned to the reference human genome (NCBI build 36.1, hg18) using Bowtie [25]. Coverage of reads mapped to the transcript was summed at each position, and the result was divided by the transcript length times the number of reads in the sample multiplied by one million.

### 4.4. Chemical Screens

PCB209 primary tumor culture was screened using a custom 60 agent target inhibitor screen denoted the Pediatric Preclinical Testing Initiative Screen Version 2.1 (PPTI screen). All screening agents were tested at either [10, 100, 1000, 10,000 nM] or [100, 1000, 10,000, 100,000 nM] based on published activity range of each compound. Compounds were purchased from third-party vendors including Selleck Chem and Sigma-Aldrich. PCB209 primary cell cultures in RPMI growth media were plated at 5000 cells/well in 384-well plates preprinted with drug. Screening plates were incubated at 37°C/5% CO_2_, for 72 hours. Cell viability was assessed by CellTiter-Glo® Luminescent Cell Viability Assay (cat. G7570, Promega, Madison, WI) following the manufacturer's protocol, and luminescence was quantified with BioTek Synergy HT plate reader (BioTek, Winooski, VT). Single agent IC_50_ values were computed via hill curve-fitting in Microsoft Excel followed by manual curation and refitting.

### 4.5. Patient-Derived Xenograft Model Development

All aspects of tissue sharing were reviewed and approved by the Oregon Health & Science University Institutional Review Board. PDX models for PCB209 were generated at JAX by implanting tumor tissue pieces into 4–6-week-old female immunodeficient NOD.Cg-*Prkdc*^*scid*^*Il2rg*^*tm1Wjl*^/SzJ (NSG) mice. When the engrafted tumor grew to ∼1000 mm^3^, the tumor was harvested and split into multiple 3–5 mm^3^ pieces and reimplanted into a new cohort of five 6–8-week-old female NSG mice for passage 1 expansion, and additional fragments were sent for quality control assessment (see below) or cryopreserved in 10% DMSO. P1 tumors that grew to 1000 mm^3^ were harvested and divided into four sections, one each for quality control, snap freezing for genomics, RNALater (Ambion) for RNA-seq, and sectioning into 3–5 mm^3^ pieces and cryopreservation in 10% DMSO.

PDX model development quality control procedures include testing the primary tumor for LCMV (lymphocytic choriomeningitis virus), bacterial contamination, and tumor cell content. The P0 and P1 tumor fragments were DNA fingerprinted using a Short Tandem Repeat (STR) assay to ensure model provenance in subsequent passages.

Immunohistochemistry (IHC) for human CD45 (IR75161-2, Agilent Technologies) was performed on engrafted tumor tissue embedded in paraffin blocks to identify lymphomagenesis. IHC for human ki67 (IR62661-2, Agilent Technologies) and vimentin (IR63061-2, Agilent Technologies) were used to ensure the propagated tumors were human rather than murine. Engrafted tumor H&E sections were reviewed by a board-certified pathologist (RGE) to evaluate morphological feature concordance between the engrafted tumor and the patient tumor.

Model information is accessible at http://tumor.informatics.jax.org/mtbwi/pdxDetails.do?modelID=TM00381.

### 4.6. Probabilistic Target Inhibition Map (PTIM) Modeling

We used PTIM modeling [16, 17, 28] to integrate PCB209 drug data with RNA sequencing data to select a personalized drug combination for PCB209.

*RNA-seq integration.* We use quantified expression data to eliminate possible false positives from chemical screen results and promote true positives among PTIM modeling targets. Here, we threshold minimum mean gene expression across the primary and PDX RNA-seq at 4 FPKM to determine potential inclusion in the PTIM computational model. RNA-seq data are integrated as below:

- *T*: drug screen targets
- *G*: drug screen targets with RNA-seq expression
- Primary*(x)*: gene expression of target *x* in primary tumor sample
- PDX*(x)*: gene expression of target *x* in explanted PDX tumor sample
- *µ*(*x*): [primary(*x*) + PDX(*x*)]/2
- ∀_*x*∈*T*∩*G*_  if *µ*(*x*) ≥ 4, keep target *x* for consideration
- ∀_*x*∈*T*∩*G*_ if *µ*(*x*) < 4, remove target *x* from consideration
- ∀_*x*∉*T*∩*G*_ keep target *x* for consideration

PTIM modeling identified the two-drug combination of ABT-737 + midostaurin as a promising combination for PCB-209. The PTIM modeling dataset is provided in Supplemental Table 6.

### 4.7. Morphoproteomic Analysis

Morphoproteomic analysis was performed by Dr. Robert Brown independent of the chemical screening, sequencing, and PTIM modeling approaches to personalized therapy assignment. Morphoproetomic analysis has been previously described in multiple publications [29–31]. Neither PTIM modeling nor chemical screening of genomic/transcriptomic data were involved in the selection of the IHC-motivated drug combination.

### 4.8. Statistics

In the PCB209 PDX experiment, the significance of variation in tumor volume with treatment was assessed with Kruskal–Wallis analysis of variance (KW ANOVA) statistical tests. All statistical testing was two-sided with a significance level of 5%.

### 4.9. Study Approval

All patients enrolled in CCuRe-FAST provided informed consent. All aspects of the study were reviewed and approved by the Oregon Health & Science University (OHSU) Institutional Review Board (IRB).

All animal procedures performed at the Jackson Laboratory were conducted in accordance with the Guidelines for the Care and Use of Laboratory Animals and were approved by the Institutional Animal Care and Use Committee at the Jackson Laboratory.

## Data Availability

High-throughput RNA sequencing data are available through the Gene Expression Omnibus (GEO, Accession ID GSE138269), and the whole genome sequencing data are available through the European Genome-Phenome Archive (EGA, Accession ID EGAS00001003981). Accessing protected data will require adhering to the requirements of the respective database systems.

## Supplementary Materials

Supplementary Materials Supplementary Table 1. Commercial ex vivo assay results and concordance—table of results transcribed from two separate commercial *ex vivo* chemosensitivity assays performed for the patient. Provides comparison and concordance of results between commercial assays for agents tested on both commercial screens. Supplementary Table 2. *Ex vivo* assay results—results of *ex vivo* sensitivity assays from commercial services and the research-level *ex vivo* targeted therapy screen. Supplementary Table 3. PCB209 drug screen results—sensitivity results from the research-level *ex vivo* targeted therapy sensitivity assay. The same drug screen was tested both on primary PCB209 cancer cells, as well as cancer cells taken from the PCB209 patient-derived xenograft (PCB209X). Supplementary Table 4. DNA and RNA sequencing data—results from DNA sequencing experiments (identifying mutations and copy number variations) and RNA sequencing experiments (quantifying gene expression) aligned across individual genes. Supplementary Table 5. Morphoproteomics—results from consultative proteomics report performed for PCB209, detailing scores, and locations of analyzed proteins from immunohistochemical experiments. Supplementary Table 6. Merged drug screen and sequencing—merged and aligned data from DNA and RNA sequencing experiments and the PCB209 primary culture and patient-derived xenograft culture research-level drug screen results, organized at the drug and gene levels. Supplementary Figure 1. Timeline of events described in this manuscript regarding patient care, commercial and research-level experiments, recurrence, and outcomes.
