# Supplementary material for: Deep Functional and Molecular Characterization of a High-Risk Undifferentiated Pleomorphic Sarcoma
Source: Sarcoma. 2020 May 4;2020:6312480. doi: 10.1155/2020/6312480 (PMC7285280; doi:10.1155/2020/6312480)
Supplement: Supplementary Materials — Supplementary Table 1. Commercial ex vivo assay results and concordance—table of results transcribed from two separate commercial ex vivo chemosensitivity assays performed for the patient. Provides comparison and concordance of results between commercial assays for agents tested on both commercial screens. Supplementary Table 2. Ex vivo assay results—results of ex vivo sensitivity assays from commercial services and the research-level ex vivo targeted therapy screen. Supplementary Table 3. PCB209 drug screen results—sensitivity results from the research-level ex vivo targeted therapy sensitivity assay. The same drug screen was tested both on primary PCB209 cancer cells, as well as cancer cells taken from the PCB209 patient-derived xenograft (PCB209X). Supplementary Table 4. DNA and RNA sequencing data—results from DNA sequencing experiments (identifying mutations and copy number variations) and RNA sequencing experiments (quantifying gene expression) aligned across individual genes. Supplementary Table 5. Morphoproteomics—results from consultative proteomics report performed for PCB209, detailing scores, and locations of analyzed proteins from immunohistochemical experiments. Supplementary Table 6. Merged drug screen and sequencing—merged and aligned data from DNA and RNA sequencing experiments and the PCB209 primary culture and patient-derived xenograft culture research-level drug screen results, organized at the drug and gene levels. Supplementary Figure 1. Timeline of events described in this manuscript regarding patient care, commercial and research-level experiments, recurrence, and outcomes. [file 6312480.f1.zip › 6312480.f1/supplementary image.pdf]

Supplementary Figures

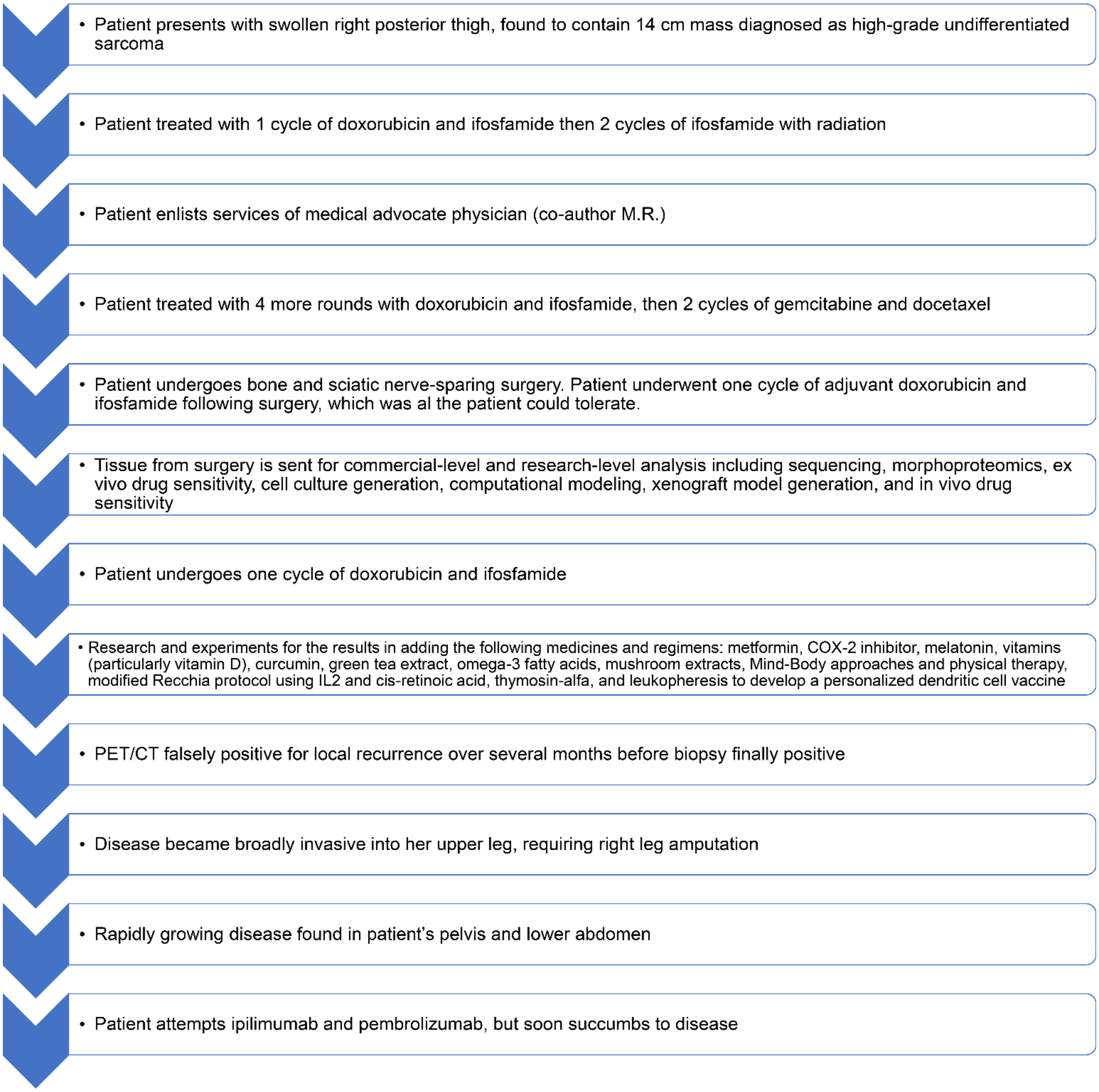

Supplementary Figure 1. Timeline of events described in this manuscript regarding patient care, commercial and research-level experiments, recurrence, and outcomes.
